# Editorial: Fertilization and early embryogenesis: from research to clinical practice

**DOI:** 10.3389/fcell.2024.1547205

**Published:** 2025-01-09

**Authors:** Martin Anger, Katerina Komrskova, Ahmed Z. Balboula, Paolo Rinaudo, Jason G. Knott

**Affiliations:** ^1^ Department of Genetics and Reproductive Biotechnologies, Veterinary Research Institute, Brno, Czechia; ^2^ Institute of Animal Physiology and Genetics, Czech Academy of Sciences, Libechov, Czechia; ^3^ Laboratory of Reproductive Biology, Institute of Biotechnology, Czech Academy of Sciences, BIOCEV, Vestec, Czechia; ^4^ Department of Zoology, Faculty of Science, Charles University, Prague, Czechia; ^5^ Division of Animal Sciences, University of Missouri, Columbia, MO, United States; ^6^ Department of Obstetrics Gynecology and Reproductive Sciences, University of California, San Francisco, San Francisco, CA, United States; ^7^ Developmental Epigenetics Laboratory, Department of Animal Science, Reproductive and Developmental Sciences Program, Michigan State University, East Lansing, MI, United States

**Keywords:** oocyte, sperm, preimplantation embryo, aneuploidy, human assisted reproduction, epigenetic regulation, placentation, maternal immune response

The fusion of an egg and a sperm triggers a complex plethora of events that will ultimately lead to the formation of a new individual. Embryonic development requires the precise orchestration of a formidable number of events. Although our knowledge of the underlying mechanisms involved has significantly increased over the last 2 decades, new discoveries continue to surprise us, further underlining the complexity and diversity of ontogenesis in different species. The intricacy of events that occur during early development, with the rapid changes in cellular morphology and behavior, may serve as an explanation for the frequent failure of mammalian embryos. This Research Topic, “Fertilization and early embryogenesis: from Research to clinical practice,” is comprised of thirteen publications that cover the complexity of germ cell, embryonic, and fetal development in a diverse number of species, including mice, cattle, pigs, humans, *Xenopus*, and axolotls. The Research Topic of these papers range from basic research on animal germ cells and embryos to clinical studies on human IVF embryos to placentation and postimplantation events.

Five studies focused on chromosomal and epigenetic aspects of early development. These include retrospective and clinical studies in humans. In Sanovec et al., the authors identified a potential new link between chromatin defects and disturbed mobility in sperm. The interaction between the DNA packaging protein protamine 2, nuclear envelope component lamin B2/3, and the cytoskeletal protein septin 12, provide a mechanistical link between abnormal sperm chromatin condensation and altered motility in a mouse model. The authors further provided evidence that a similar connection exists in human sperm. For instance, the altered expression and localization of homologue proteins is associated with low sperm motility known as asthenozoospermia.

Embryonic development is known to be prone to errors during chromosome segregation. In the study by Horakova et al., the authors showed that the spindle assembly checkpoint (SAC), a surveillance mechanism that ensures accurate chromosome attachment to spindle microtubules prior to cell division, is not utilized by early mouse embryos. Instead, the blastomeres of 2-cell embryos activate a complex known as the anaphase promoting complex (APC/C) immediately after nuclear envelope breakdown (NEBD). Therefore, the absence of SAC may partially explain the higher frequency of aneuploidy in embryos.

In the study by Ma et al., the authors recruited two female patients from a family characterized by recurrent early embryo failure after IVF or ICSI. Using whole-exome sequencing on zygotes, the authors identified a mutation in a gene that encodes for regulator of G protein signaling 12 (RGS12). Phenotypic analysis of oocytes and zygotes from these patients revealed defects in calcium signaling, prolonged CSF arrest, and the inability to activate APC/C sufficiently, resulting in arrest after the 1-cell stage. The authors postulate that the RGS12 gene mutation plays a causal role in recurrent early embryo failure.

Human IVF embryos can exhibit unusual patterns of cell division after IVF or ICSI such as fast cell (FC) division to the 3-cell stage via two consecutive divisions or instant direct cleavage (IDC) into 3 blastomeres. The utilization rate of embryos that exert these types of cleavage is not known. In a retrospective study by Nemerovsky et al., the authors show that although FC dividing embryos exhibited reduced development into blastocysts, the pregnancy rate was similar to controls. Most IDC embryos arrested on day 3, and those that developed into blastocysts did not produce a pregnancy. Since blastomeres resulting from fast division might be aneuploid, this illustrates that developing human embryos exhibit a surprising degree of plasticity and redundancy.

The review by Montgomery et al. provides an exquisite overview of the developmental dynamics of DNA methylation and the role of ten-eleven translocation (TET) enzymes in demethylation in oocytes, preimplantation embryos, primordial germ cells, and adults. These enzymes are not only essential for epigenetic reprogramming during normal development, but their malfunction is associated with various types of cancer and developmental disorders. The authors further discuss the implications of TET enzymes in human ART.

Two studies focused on the microscopic assessment of oocytes and preimplantation embryos and the impact of oil-covered culture media on the efficacy of small molecule inhibitors. Oocyte and embryo quality is paramount for their utilization in ART. However, it is not always possible to assess oocytes and early embryos without compromising the developmental potential. Thanks to the recent progress in live imaging and micromanipulation, the biomechanical properties of oocytes and embryos can be assessed using noninvasive techniques. Fluks et al. provide a comprehensive review on the biomechanical properties of oocytes and preimplantation embryos and discuss various techniques that are used to evaluate these properties.

In the laboratory it is a common practice to use small molecule inhibitors to block the activity of key cell-cycle regulators and signaling proteins in oocytes and preimplantation embryos. In Rémillard-Labrosse et al., the authors demonstrated that several different inhibitors lose activity in standard oil-covered culture media, likely by partitioning into the oil. The authors conclude that researchers should be extremely cautious when using oil to culture oocytes and embryos in the presence of small molecule inhibitors and recommend using oil-free culture systems.

Three studies focused on embryonic development and tetraploid complementation. In Šimková et al., the authors assessed spatiotemporal changes in expression of various transcripts during early development in axolotl. Since the localization of specific transcripts is essential for the development of the body plan, the comparison was aimed at identifying similarities and differences between axolotls and *Xenopus*. The authors reported surprising differences between both species. One notable difference was the development of primordial germ cells, indicating that even fundamental processes might not be conserved between closely related species.

In Kang et al., the authors focused on the effect of growth differentiation factor 8 (GDF-8) on the *in vitro* development of bovine oocytes and embryos. The authors demonstrated that the supplementation of culture media with GDF-8 improved embryo quality by increasing the total number of cells in blastocysts and enhancing the expression of multiple transcripts, including mRNAs that encode for tight junction proteins important for blastocyst cavitation. The authors further show that cryopreserved GDF-8 treated blastocysts recovered and re-expanded better than untreated embryos.

Tetraploid complementation is highly efficient method for testing the developmental potential of pluripotent cells. Lee et al. compared various methods for producing tetraploid embryos in pigs and discovered that tetraploids produced by the electrofusion of 2-cell blastomeres in parthenogenetic embryos, exhibited the greatest developmental potential with significantly lower rates of apoptosis compared to the other methods tested.

Lastly, three papers focused on the role of the maternal immune system during pregnancy, the importance of *in vitro* models for placental research, and research on fetal steroidogenesis. It is well known that the functional modifications of the maternal immune system are essential for successful embryonic and fetal development. However, we still do not fully understand how immune tolerance of the sperm and embryo is established in the reproductive tract while the immune system is still able to react to most pathogens. Recent literature on this subject is elegantly reviewed by Visnyaiová et al. The authors provide an extensive overview on the diverse population of immune cells that occupy the uterine tube. They discuss the possibility of immune privilege as a mechanism to tolerate sperm and embryos in the uterine tube. The authors conclude that more studies concerning the specificity of the maternal immune system and its interaction with the developing embryo and fetus are needed.

A prerequisite for a successful pregnancy is the establishment of a healthy placenta, a transient organ that supports the growth and health of the fetus. Alterations in placentation or placental function can have a profound impact on embryonic and fetal development and the health of the offspring. In Liu et al., the authors provide an expansive review on the biology of the human placenta and the available *in vitro* models used for studying human placentation and placental function. Examples of these models include placental-derived trophoblastic stem cell (TSC) models, primary trophoblast cells, and organoid models. The advantages and limitations of each model are discussed.

In adults, it is well established that testosterone is produced by Leydig cells in part through the actions of the enzyme 17β-hydroxysteroid dehydrogenase (HSD17B3). However, the cell-specific expression and localization of HSD17B3 during human fetal development is not as clear. In Planinic et al., the authors used a unique cohort of human fetal testes samples to follow the expression and localization of HSD17B3. They traced its origin to the Sertoli cells during the second trimester, which is similar to the Sertoli cell-specific expression observed in rodents. The authors postulate that perturbations in the transition of steroidogenesis from Sertoli cells to Leydig cells could be a potential source of problems in the development of the testes and future sperm production.

